# Rigid Polyurethane Foams Based on Bio-Polyol and Additionally Reinforced with Silanized and Acetylated Walnut Shells for the Synthesis of Environmentally Friendly Insulating Materials

**DOI:** 10.3390/ma13153245

**Published:** 2020-07-22

**Authors:** Sylwia Członka, Anna Strąkowska

**Affiliations:** Institute of Polymer and Dye Technology, Faculty of Chemistry, Lodz University of Technology, Stefanowskiego 12/16, 90-924 Lodz, Poland; anna.strakowska@p.lodz.pl

**Keywords:** rigid polyurethane foams, lignocellulosic materials, filler, chemical treatment, mechanical characteristics

## Abstract

Rigid polyurethane (PUR) foams produced from walnut shells-derived polyol (20 wt.%) were successfully reinforced with 2 wt.% of non-treated, acetylated, and silanized walnut shells (WS). The impact of non-treated and chemically-treated WS on the morphology, mechanical, and thermal characteristics of PUR composites was determined. The morphological analysis confirmed that the addition of WS fillers promoted a reduction in cell size, compared to pure PUR foams. Among all the modified PUR foams, the greatest improvement of mechanical characteristics was observed for PUR foams with the addition of silanized WS—the compressive, flexural, and impact strength were enhanced by 21, 16, and 13%, respectively. The addition of non-treated and chemically-treated WS improved the thermomechanical stability of PUR foams. The results of the dynamic mechanical analysis confirmed an increase in glass transition temperature and storage modulus of PUR foams after the incorporation of chemically-treated WS. The addition of non-treated and chemically-treated WS did not affect the insulating properties of PUR foams, and the thermal conductivity value did not show any significant improvement and deterioration due to the addition of WS fillers.

## 1. Introduction

Nowadays, polyurethanes (PUR) are used in many areas, such as thermal insulation materials, automotive elements, construction parts, medical devices as well as in the production of elastomers, adhesives, and foams [1]. PUR are synthesized by a reaction between hydroxyl groups (–OH) and isocyanate groups (–NCO). Due to the non-biodegradability and high toxicity, both petrochemical-based compounds have a negative impact on the environment [2,3]. Due to this, bio-based polyols from sustainable raw materials, such as plant oils, have attracted great interest in the synthesis of environmentally-friendly PUR foams [4,5,6,7]. Bio-polyols derived from certain types of cellulosic sources, such as spent coffee [8], cassava residue [9] or jute fibers [10] have been examined for the synthesis of PUR materials. In our previous work, PUR foams were prepared using the bio-polyol derived from lignocellulosic walnut shells (WS) [11]. It has been shown that due to the rich organic nature (~50% of lignin, ~24% of cellulose and ~24% of hemicellulose) [12,13], WS can be successfully applied for the production of bio-polyols for the synthesis of PUR foams. Therefore, liquefaction of WS has been conducted using a mixture of glycerine and polyethylene glycol (PEG-400) in the presence of acid catalyst (sulphuric acid). The impact of WS-based polyol on the mechanical and thermal characteristics of PUR foams have been examined. The results showed that it is possible to convert these lignocellulosic residues into polyol and produce PUR foams with the properties somewhat similar to those of commercial foams, although with higher thermal conductivity. 

Unfortunately, a common phenomenon observed after the synthesis of PUR foams from bio-polyols is the deterioration of mechanical parameters of these materials [11]. Many previous studies have shown that the incorporation of a selected amount of fillers with both, organic or inorganic nature, helps to improve the mechanical characteristic of polymeric composites [14,15]. Therefore, to improve the mechanical characteristic of PUR foams, an additional fillers in the form of nanoparticles or fibers are incorporated. For example, PUR foams modified with a ground waste of bulk moulding composites were successfully synthesized by Barczewski et. al. [16]. When compared with unmodified PUR foams, the addition of inorganic filler resulted in the formation of composites with improved thermo-mechanical performances and better fire retardancy. Zhou et al. [17] have synthesized PUR composites from palm oil-based polyol. The bio-composites were additionally modified with the addition of selected amounts of cellulose nanocrystals. The resulting composites exhibited improved mechanical performance and better dimensional stability under the selected temperatures. Rigid PUR composites synthesized from bio-polyol and additionally enhanced with paper waste sludge (PWS) particles were synthesized by Kairyte et. al. [18]. The addition of 5 wt.% of PWS resulted in the production of PUR foams with better water vapor resistance, higher density, and improved mechanical characteristics, such as compressive strength and elastic modulus. Paciorek-Sadowska et al. [19] synthesized PUR foams with rapeseed cake as a natural filler. The addition of 30-60 wt.% of rapeseed cake filler resulted in the production of PUR composites with higher density, greater mechanical performances, and improved fire resistance. 

Besides, many lignocellulosic fillers can be successfully used as reinforcing materials in polymer matrices [20,21,22,23,24,25,26,27], it was reported, that their hydrophilic nature may limit their further application. Due to this, the surface modification of the fillers seems to be a sufficient step before the application of natural fillers as reinforcing materials for polymeric composites [28,29,30,31]. Previous studies have proved that chemical modifications of the filler surface, such as acetylation [32], alkalization [33], benzoylation [34], and silanization [35] may successfully improve the adhesion between the filler and the polymeric matrix. Although many published studies are devoted to the examination of PUR composites, just a few research has been done on the surface treatment of WS and the impact of acetylated and silanized WS on selected properties of PUR foams. Therefore, in this study, the effect of acetylated and silanized WS on the morphological, mechanical, insulating, and thermal characteristics of the PUR composites was investigated.

## 2. Experimental Section

### 2.1. Chemicals and Materials

Commercial polyester polyol with a brand name STEPANPOL PS-2352 and aromatic diisocyanate with a brand name PUROCYN B were purchased from Purinova Sp. z o.o (Bydgoszcz, Poland). Silicone surfactant with a brand name TEGOSTAB B8513 and PUR metal catalysts-Kosmos 33 (potassium acetate) and Kosmos 75 (octoate catalyst) were provided by Evonik Industries AG (Essen, Germany). Cyclopentane (blowing agent) and pentane (blowing agent) were purchased from Merck KGaA (Darmstadt, Germany). Sodium hydroxide (anhydrous), acetic acid (≥99.9%), ethanol (≥99.9%), sulfuric acid (purity 95–98%) were provided by Sigma-Aldrich Corporation (Saint Louis, MO, USA). Triphenylsilanol was provided by abcr GmbH Company (Karlsruhe, Germany). Walnut shells were kindly provided by a Polish local company (Lodz, Poland).

### 2.2. Methods

#### 2.2.1. Pre-Treatment of WS with an Alkali Solution

WS filler was pretreated with 10% NaOH solution. A calculated amount of WS filler was soaked in the NaOH solution for 1 h. After that, the solution was neutralized with 1% acetic acid. Such obtained alkali-treated filler was washed with ultrapure water to pH of 7 and dried in an oven (24 h, 80 °C).

#### 2.2.2. Silanization of WS

Alkali-treated WS filler was treated with a 5% triphenylsilanol solution. The calculated amount of WS filler was soaked in a solution of triphenylsilanol in ethanol, maintaining the ratio of WS filler to a solution at the level of 1:20 (by weight). After 3 h, the triphenylsilanol solution was evaporated, and the silane-treated WS filler was separated from the solvent. The silane-treated WS was washed with ultrapure water and dried in an oven (24 h, 80 °C).

#### 2.2.3. Acetylation of WS

Alkali-treated WS was soaked in a mixture of acetic acid and acetic anhydride (1:1 *v*/*v*). To promote the reaction, a few drops of sulfuric acid were dropped into the mixture and the solution was intensively stirred for 30 min. Lastly, the WS was removed from the mixture, thoroughly washed with ultrapure water to pH of 7. The acetylated WS filler was placed in an oven (120 °C) and dried, until a constant weight of the WS filler was obtained.

#### 2.2.4. PUR Foams Preparation

**Figure 1:**
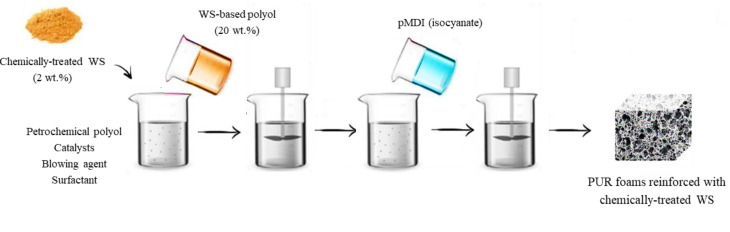
Schematic procedure of PUR foam synthesis.

The selected amounts of polyester polyol, WS-based polyol, surfactant, catalysts, flame retardant, and blowing agent were placed in a cylindrical form and mixed with mechanical stirring (1000 rpm, 30 s). The obtained mixture was modified with the addition of 2 wt.% of non-treated, silanized, or acetylated-WS, and thoroughly mixed at 1000 rpm for 60 s. After the complete dispersion of WS filler, an isocyanate component was poured into the mixture and stirred for another 30 s. The resulting PUR foams were expanded freely in open forms. Before further characterization, PUR foams were conditioned at a standardized temperature of 25 °C and humidity of 50% for 24 h. The detailed formulations presenting the weight ratio of components are shown in Table 1. The schematic procedure of PUR foams synthesis is given in Figure 1.

### 2.3. Test Methods

The dynamiclLight scattering (DLS) method was used to evaluate the average size of filler particles using a Zetasizer NanoS90 instrument (Malvern Instruments Ltd, Malvern, UK). WS particles were dispersed in a polyol (0.04 g·L^−1^) and the average of 5 individual measurements was evaluated.

The viscosity of PUR systems was determined according to ISO 2555 [36]. The measurement was performed using a rotatory viscometer (Viscometer DVII+, Brookfield, Berlin, Germany). The viscosity of PUR systems was determined at different share rates—0.5, 5, 10, and 100 rpm (round per minute). The average of 5 individual measurements was evaluated. The standard deviation was calculated.

The chemical structure of fillers was determined by Fourier-transform infrared spectroscopy (FTIR, Nicolet iS50 spectrometer, Thermo Fisher Scientific, Waltham, MA, USA). An average of 64 individual scans was evaluated.

Scanning electron spectroscopy (JEOL JSM-5500 LV, JEOL Ltd., Peabody, MA, USA) was selected to analyze the structure of the fillers and PUR foams. The morphology features of PUR foams were evaluated using ImageJ software (Java 1.8.0, Media Cybernetics Inc., Rockville, MD, USA).

The PUR foam density was calculated as the ratio of PUR mass to volume, following ISO 845 [37]. The average of 5 individual measurements was evaluated. The standard deviation was calculated. 

Compressive strength (σ_10%_), flexural strength (ε_f_), and impact test were processed using Zwick Z100 Testing Machine (Zwick/Roell Group, Ulm, Germany), following ISO 844 [38], ISO 178 [39], and ISO 180 [40], respectively.

Thermogravimetric analysis (TGA) and differential thermogravimetry (DTG) were applied to determine the thermal properties of PUR samples. A thermal analysis test was conducted in an argon atmosphere using STA 449 F1 Jupiter Analyzer (Netzsch Group, Selb, Germany). The samples with an initial weight of 10 mg were examined in the selected range of temperatures (from 0 to 600 °C).

The dynamic-mechanical characteristic (DMA) was performed using a modular compact rheometer (ARES, TA Instruments, New Castle, DE, USA) under the selected parameters (constant deformation of 0.1%, frequency of 1 Hz). The PUR samples were examined in the selected range of temperatures (from 0 to 250 °C). The average of 5 individual measurements was evaluated. The standard deviation was calculated.

The thermal conductivity of the PUR foams was examined using the LaserComp 50 heat flow meter (HFMA, Westchester, IL, USA). The average of 5 individual measurements was evaluated. The standard deviation was calculated.

## 3. Results and Discussion

### 3.1. Characterization of WS-Based Polyol

The properties of WS-based polyol have been widely discussed in our previous study [11]. The selected properties of bio-polyol and polyester polyol (STEPANPOL PS-2352) are shown in Table 2. 

### 3.2. Characterization of WS Filler

**Figure 2:**
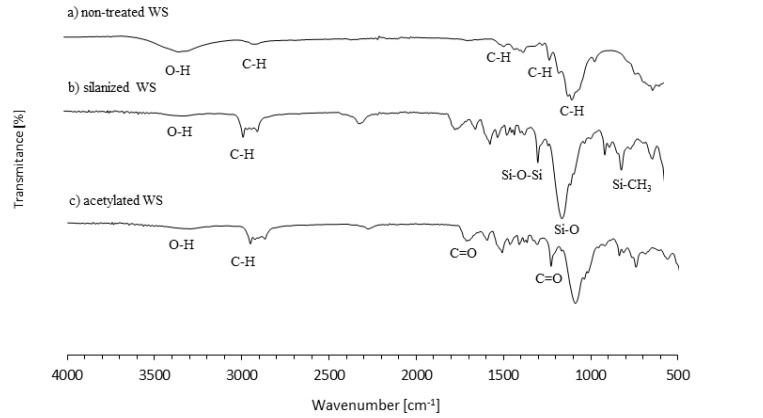
Fourier-transform infrared spectroscopy (FTIR) spectra of (**a**) non-treated WS, (**b**) silanized WS, and (**c**) acetylated WS.

The chemical structure of non-treated, acetylated, and silanized WS was examined using FTIR analysis. The obtained spectra are presented in Figure 2. Bands located at 840, 1035, 1455, and 2900 cm^−1^ are characteristic for the C–H vibration of cellulose, hemicellulose, and lignin of WS, respectively [41]. The new band at ~740 refers to Si–CH_3_ vibration and confirms the formation of chemical linkage between the silane coupling agent and WS filler [42]. Other bands characteristic for silanized WS occur at 1340, 1080, 3350 cm^−1,^ and refer to Si–O–Si, Si–O, and O–H vibration of silanized WS, respectively [42,43]. The acetylation of WS is confirmed by a reduced intensity of band attributed to OH vibration that occurs at 3300 cm^−1^ [20,44]. Moreover, the presence of new peaks at 1230 cm^−1^ and 1730 cm^-1^, which are characteristic for C=O stretching of the ester carbonyl group and confirms the successful acetylation of WS filler [45,46,47].

**Figure 3:**
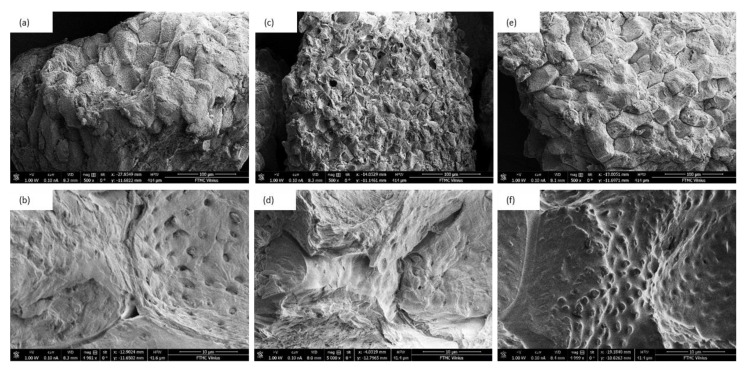
SEM results of (**a**,**b**) non-treated WS (**c**,**d**) acetylated WS, and (**e**,**f**) silanized WS.

Silanization and acetylation treatments affect the morphology of the WS (Figure 3). The surface of non-treated WS is rough and some cracks are visible on the filler surface. After the silanization and acetylation, the topography of the WS filler becomes rougher, because the chemical treatments, such as alkalization, may remove the waxy substances that smooth the filler surface [48,49,50,51]. However, the previous studies have reported that such a rough topography of the filler particles may have a beneficial effect on the further mechanical properties of composites [52,53]. The rough surface of the filler may improve the mechanical interlocking and interphase bonding between the filler surface and polymeric phase, which, in turn, results in better mechanical characteristics of such reinforced composites.

**Figure 4:**
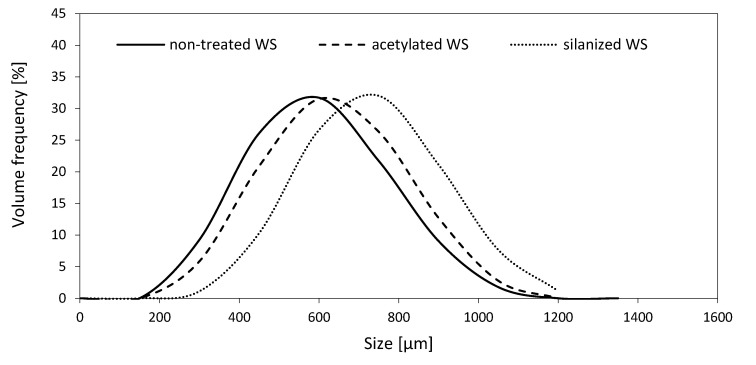
The results of particle size distribution.

The size of WS’s particles was examined in polyol dispersion. The particle size distribution of the non-treated, acetylated, and silanized WS are given in Figure 4. The presented results indicate that the size of non-treated and chemically-treated WS ranges from 300 to 1000 µm in all cases. The highest percentage of particles for non-treated WS, acetylated WS, and silanized WS is observed at ~600, ~650, and ~700 µm, respectively.

As presented in Table 3, comparing to PUR_0, the addition of WS filler results in a greater viscosity. This may be connected with the Van der Waal’s forces and hydrogen bonding that occur between the active groups of WS particle and polyester polyol [54]. With the addition of non-treated WS, acetylated WS, and silanized WS, the viscosity increases from 840 mPa·s to 1700, 1900, and 2050 mPa·s, respectively. All PUR mixtures behave as non-Newtonian fluids, and reveal an analog tendency to the PUR mixtures containing various kinds of organic and/or inorganic fillers, which were reported in previous works [36,37].

### 3.3. Foaming Kinetic

The impact of WS filler on the foaming process of PUR foams was monitored by measuring the duration of the cream time, expansion time, and tack-free time. The cream time refers to the rise start of the PUR system, the expansion time refers to the transition of the liquid-state to solid-state, and tack-free time is measured until the foam solidifies completely. The results of characteristic times are presented in Table 4.

Due to the incorporation of non-treated, acetylated, and silanized WS, the cream and rise times have increased [55,56,57]. In the case of PUR foams containing WS filler, the extended expansion time may be connected with a limited expansion of cells. Due to the increased viscosity of the PUR system, the mobility of the molecular chains is reduced, which, in turn, affects the polymerization kinetic of PUR synthesis. As the viscosity of the PUR mixtures increases, the mass transfer of the blowing agent from the solid to the gas phase decreases, and the expansion of PUR foam is slowed down [55,56,57]. Similar results have been shown by Kurańska et al. [58]. The authors confirmed that the incorporation of the vegetable fillers, such as wood fibers, results in the elongation of processing times due to the reduced reactivity of the modified systems.

### 3.4. Cellular Structure and Thermal Conductivity

**Figure 5:**
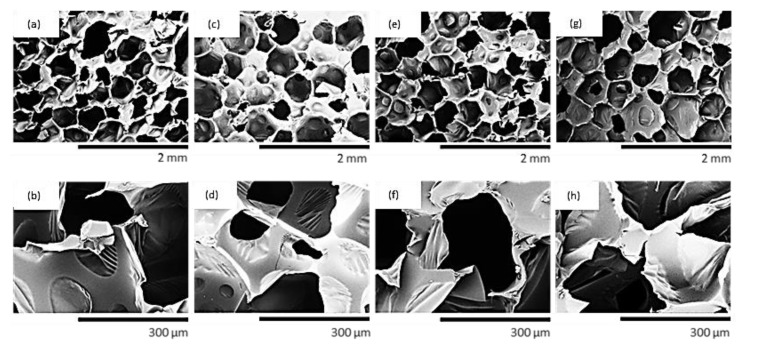
Cellular structure of (**a**,**b**) PUR_0, (**c**,**d**) PUR_WS_NT, (**e**,**f**) PUR_WS_A and (**g**,**h**) PUR_WS_S.

The impact of non-treated WS and chemically-treated WS on the cellular structure of PUR foams was examined using SEM (Figure 5). Mean cell size and closed-cell content are listed in Table 5. When compared with neat PUR_0, the incorporation of each filler leads to a decrease of average cell size, i.e., 380, 360, and 355 μm for PUR_WS_NT, PUR_WS_A, PUR_WS_S against 410 μm for neat PUR_0. Based on this result, it can be concluded that WS particles can promote the nucleation of the air bubbles, and prevent their coalescence during the expansion process. Paciorek-Sadowska et al. [19] have reported a similar tendency—at a higher loading of rapeseed cake, the cellular structure of PUR composites was more heterogeneous, and a reduced cell diameter was observed—for example, the incorporation of 60 wt.% of rapeseed cake has reduced an average diameter from 316 to 250 µm.

Comparing the PUR foams containing non-treated and chemically-treated PUR foams. it can be seen that the incorporation of silanized and acetylated WS filler leads to the production of PUR foams with regular structure and more uniform cells. Moreover, the incorporation of non-treated and chemically-treated WS affects the closed-cell content of the foams. When compared with PUR_0, the closed-cell content decreases slightly after the incorporation of WS filler, and this trend is more visible in the case of PUR composites containing non-treated WS filler. Thus, the chemical treatment of WS can improve the interphase adhesion between filler particles and polymeric matrix, which leads to the production of PUR composites with a more stable cellular morphology. A cross-linked structure of PUR containing filler particles can prevent the collapse of the cells during their expansion and form additional edges that are able to capture the emitted CO_2_ [59]. Sung et al. [60] stated that an increase in pore size is connected with the hydrophobic character of the filler surface-hydrophobic fillers exhibit greater adhesion to the polymer matrix, while the higher the hydrophilicity, the lower the adhesion and greater the cell size of the foam structure. Such a tendency may be also found in our study. Among the examined PUR foams, PUR_WS_NT is characterized by larger cell diameters, due to the highly hydrophilic nature of WS. Acetylation and silanization treatments increase the hydrophobic character of WS, thus the obtained PUR composites are characterized by more uniform cells with reduced diameters. 

**Figure 6:**
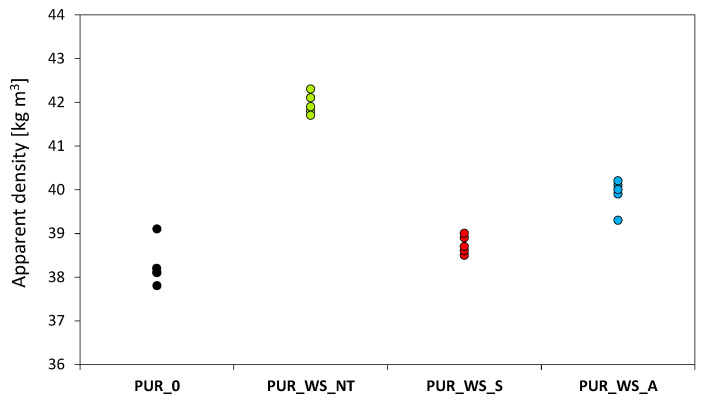
The apparent density of PUR_0, PUR_WS_NT, PUR_WS_S, and PUR_WS_A.

The impact of WS on the density of PUR foams is presented in Figure 6 (for each measurement, the entire experiment was repeated, and the presented value concerns the individual samples). Comparing to PUR_0, the addition of WS fillers increases the density of PUR composites. The average density of the neat PUR_0 is 38 kg m^−3^, while the density of modified PUR foams oscillates between 39 and 42 kg m^−3^ upon incorporating WS filler. It is clear that non-treated and chemically-treated WS affect the value of apparent density differently, due to their different dispersion in the PUR matrix. The higher level of dispersion, associated with the chemical modification of WS leads to lower nucleation energy and greater adhesion between WS filler and PUR matrix, which determines a finer cell morphology and lower apparent density [61]. As a result of this, PUR_WS_A and PUR_WS_S exhibit lower density than PUR_WS_NT.

Thermal conductivity (λ) is an important parameter that determines the insulation properties of PUR foams [16,62,63]. Generally, the λ value of PUR foams is calculated as a sum of λ of the gas in the cells (λgas), the λ through the solid polymer (λsolid), the radiation heat transfer across the walls of the solid struts (λradiation), and the convection of the gas within the cells (λconvection) [64]. As presented in Table 5, thermal conductivity for PUR_0 is 0.0251 W m^−1^K^−1^. The incorporation of non-treated WS increases the value of λ to 0.0302 W m^−1^K^−1^, however, it remains almost unchanged for PUR_WS_A and PUR_WS_S—the value of λ increases slightly to 0.0293 and 0.0284 W m^−1^K^−1^, and it is still in line with the conditions of insulation materials [1,65].

### 3.5. Mechanical Characteristics

**Figure 7:**
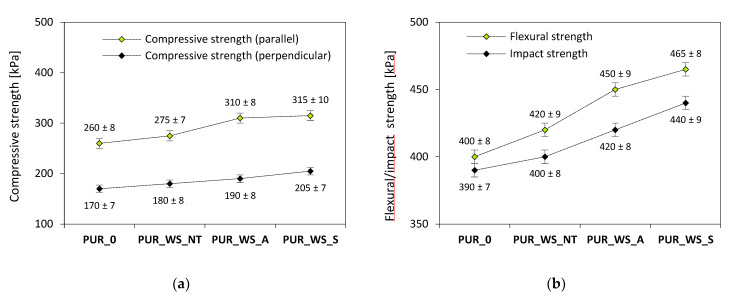
(**a**) Compressive, (**b**) flexural and impact strength of PUR foams.

Compressive strength (σ_10%_), flexural strength (σ_f_), and impact strength were examined to determine the impact of surface WS fillers on the mechanical characteristics of PUR composites. The results of σ_10%_ are presented in Figure 7. When compared with PUR_0, σ_10%_ increases by ~6% for PUR_WS_NT. The mechanical strength increases after the chemical modifications of WS filler-the value of σ_10%_ increases by ~19% and ~21% for PUR_WS_A and WS_WS_S, respectively. Such a result can be connected with the cellular structure of the resulting composites, which has a great impact on their further mechanical properties. As discussed previously, PUR foams containing acetylated and silanized WS have a more uniform structure, with a greater number of closed-cells (see Figure 5). Moreover, after the silanization and acetylation treatment, the possible chemical reaction may occur between the hydroxyl groups of WS and acetic acid or silane coupling agent, creating chemical covalent linkages that improve the interfacial adhesion between the filler and the polymeric matrix [66,67]. Ciobanu et al. [68] reported that the incorporation of rigid, three-dimensional lignin improved the mechanical characteristics of the resulting products. This was attributed to the fact that incorporated lignin may act as a compatibilizer for PUR segments, successfully enhancing the mechanical properties of the resulting composites. 

The results of *σ_f_* are shown in Figure 7. After the incorporation of non-treated, acetylated, and silanized WS the value of *σ_f_* is increased. When WS filler is incorporated without treatment, the *σ_f_* increases by ~5% when compared with PUR_0. A further improvement is observed for PUR_WS_A and PUR_WS_S—the value of *σ_f_* increases by ~12% and ~16%, respectively. As followed by flexural properties, the impact strength was measured as well (Figure 7). When comparing with PUR_0, the impact strength of PUR foams containing non-treated and chemically-treated WS is increased. The greatest improvement is observed for PUR_WS_S—the impact strength increases by ~13%. Previous studies have shown that the finer cellular structures can improve the mechanical characteristics of porous materials, thus, the PUR foams containing acetylated and silanized WS have a greater flexural strength [69,70]. Moreover, the chemical modification of the filler may result in the rough morphology of that promotes the mechanical interlocking between the filler surface and polymeric matrix, enhancing the mechanical characteristics of the resulting composites. The rigid cellular structure of PUR foams containing solid particles of the filler is able to absorb more energy during impact, resulting in the better mechanical behavior of PUR foams [19].

### 3.6. DMA Results

**Figure 8:**
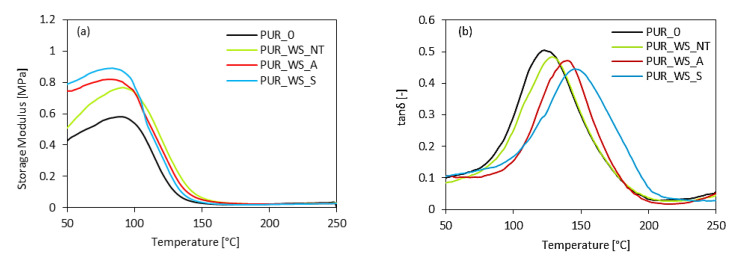
(**a**) Storage modulus and (**b**) tanδ of PUR foams.

DMA results are given in Figure 8. The storage modulus (E’) of the PUR foams containing non-treated, acetylated, and silanized WS is higher than the PUR_0. This indicates a reinforcing effect of the WS filler, which results in effective stress transfer from the filler to the PUR matrix. As observed in Figure 8a, PUR_WS_A, and PUR_WS_S have a higher value of E’ than that containing non-treated PUR_WS_NT. As reported in previous works, the chemical modification removes waxes and surface impurities from cellulose fillers, thus, increasing its interaction with the polymer matrix [48,49,50,51]. Additionally, the chemical treatment helps in the fibrillation of the filler surface. Therefore, it can be concluded that the rough morphology of the WS filler may improve the mechanical interlocking between the filler and polymeric matrix, which, in turn, results in more efficient stress transfer from the matrix to the filler particles and increased storage modulus.

The glass transition temperature (T_g_) of PUR foams refers to the maximum of tanδ in the function of the temperature (Figure 8b). All series of PUR foams show one peak of maximum on the graph, indicating that the resulting composites can be classified as homogeneous blends. Moreover, PUR foams containing WS filler exhibit higher value of T_g_, which may be connected with higher viscosity of PUR systems and reduced molecular mobility of polymer chains. As the T_g_ values of the treated PUR_WS_A and PUR_WS_S are higher than non-treated PUR_WS_NT, it can be concluded that chemical treatment affects the WS surface and improves the interphase contact between the filler and PUR matrix. A lower tanδ peak value for PUR_WS_NT may be connected with weaker linkages between the filler surface and the polymeric matrix, which results in a greater dissipation of energy. Since the tanδ peak value illustrates the filler matrix adhesion, it can be concluded that the higher tanδ peak value observed for PUR_WS_A and PUR_WS_S confirm the better compatibility between filler surface and the polymeric matrix.

### 3.7. Thermal Stability

**Figure 9:**
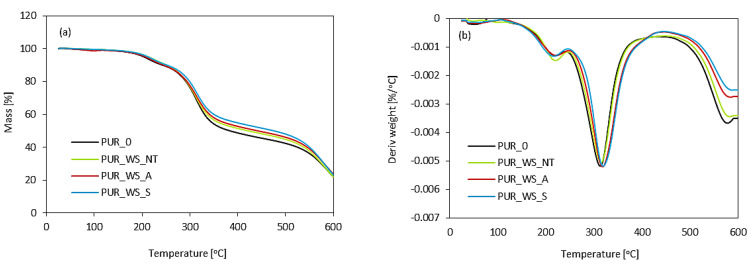
(**a**) TG and (**b**) differential thermogravimetry (DTG) curves obtained for PUR foams.

TGA results are given in Figure 9 and Table 6. Results showed improved thermal characteristics of PUR composites containing chemically-treated WS, however, there is almost no change in the case of non-treated PUR_WS_NT.

The first degradation step refers to the temperatures of 5% weight loss (T_5%_). It was noticed that the value of T_5%_ increases slightly from 220 °C (for PUR_0) to 224 and 225 °C by the incorporation of acetylated and silanized WS, respectively, while there is almost no change for PUR_WS_NT. 

The second stage of degradation refers to the thermal decomposition of soft segments of PUR and occurs in the range of 300–350 °C [71]. For PUR_0, the second stage is observed at 315 °C. A slight improvement in thermal stability exhibits in PUR_WS_A and PUR_WS_S; when compared with PUR_0, the temperature is increased by 8 and 10 °C, respectively. Such an improvement can be connected with a greater crosslink density, due to the addition of acetylated and silanized WS. A more rigid structure of PUR composites may successfully limit the mobility of the polymeric chains, which, in turn, reduces heat transport and improves the thermal stability of PUR composites [72].

The third degradation step of PUR foams in observed in the range of 500–600 °C. The third degradation step is mostly connected with the degradation of organic compounds incorporated into the PUR matrix, such as cellulose, hemicellulose, and lignin [73]. The thermal characteristic of PUR foams containing WS filler is similar to those of the unmodified PUR foam (PUR_0). With the incorporation of acetylated and silanized WS, the mass loss increases due to the presence of cellulose [72]. The char residue mass is another factor that determines the thermal stability of PUR foams. It can be seen that, after the incorporation of each kind of WS filler, the amount of char residue is increased. For PUR_0, the char residue is 48.1% at 450 °C, while, for PUR foams containing non-treated and chemically-treated WS, the amount of char residue increases to 49.8% (for PUR_WS_NT), 50.2% (for PUR_WS_A), and 51.5% (for PUR_WS_S). The rigid structure of lignin can limit the heat transport through the PUR foam structure, which results in better thermal stability of PUR composites [73]. Analog results have been shown in previous studies as well [74,75].

## 4. Conclusion

In the present work, the impact of non-treated, acetylated, and silanized WS on morphological, mechanical, thermal, and physical properties of PUR foams has been investigated and discussed. It has been shown that the cellular structure was affected, due to the addition of non-treated and chemically-treated WS. PUR composites with the addition of acetylated and silanized WS exhibited a more uniform structure than PUR foams with the addition of non-treated WS. The mechanical characteristics of PUR foams, such as compressive, flexural, and impact strength, were improved after the incorporation of non-treated and chemically-treated WS. Among all the specimens, the greatest improvement was observed for PUR foams containing silanized WS—the compressive, flexural, and impact strength were improved by 21, 16, and 13%, respectively. From this investigation, it can be concluded that the acetylation and silanization of WS can improve the interfacial bonding between WS and PUR matrix, enhancing the mechanical properties of the PUR foams. Moreover, the addition of non-treated and chemically-treated WS improved the thermal properties of PUR foams—e.g., at 600 °C, the char residue increased from 21.9 to 25.5% for PUR foams, with the addition of acetylated WS. The addition of WS fillers did not affect the thermal conductivity of PUR foams.

## Figures and Tables

**Table 1:** The weight ratio of components used for polyurethanes (PUR) foam synthesis.

Component	PUR_0	PUR_WS_NT	PUR_WS_A	PUR_WS_S
Amount, parts by STEPANPOL PS-2352 weight [pbw]				
STEPANPOL PS-2352	80	80	80	80
WS-based polyol	20	20	20	20
PUROCYN B	160	160	160	160
Kosmos 75	6	6	6	6
Kosmos 33	0.8	0.8	0.8	0.8
Tegostab B8513	2.5	2.5	2.5	2.5
Water	0.5	0.5	0.5	0.5
Pentane/cyclopentane	11	11	11	11
Non-treated WS	0	2	0	0
Acetylated WS	0	0	2	0
Silanized WS	0	0	0	2

**Table 2:** Selected properties of polyester polyol and walnut shell (WS)-based polyol [11].

Component	Viscosity [mPa s]	Molecular Weight (M_w_) [Da]	Hydroxyl Number [mg KOH/g]
WS-based polyol	2550	420	340
STEPANPOL PS-2352	2000-4500	468	230–250

**Table 3:** Dynamic viscosity of PUR systems.

Sample	Dynamic Viscosity η [mPa·s]			
	0.5 rpm	5 rpm	10 rpm	100 rpm
PUR_0	840 ± 10	520 ± 8	470 ± 9	230 ± 7
PUR_WS_NT	1700 ± 12	1200 ± 10	750 ± 9	450 ± 8
PUR_WS_A	1900 ± 11	1300 ± 10	1050 ± 10	580 ± 8
PUR_WS_S	2050 ± 10	1450 ± 12	1200 ± 11	650 ± 7

**Table 4:** Processing times of PUR foams.

Sample	Cream Time [s]	Expansion Time [s]	Tack-Free Time [s]
PUR_0	56 ± 3	419 ± 8	340 ± 7
PUR_WS_NT	60 ± 1	495 ± 9	375 ± 8
PUR_WS_A	59 ± 2	510 ± 6	365 ± 9
PUR_WS_S	59 ± 3	515 ± 8	370 ± 8

**Table 5:** Structural parameters and thermal conductivity results of PUR foams.

Sample	Cell Size [µm]	Apparent Density [kg m^−3^]	Closed-Cell Content [%]	Thermal Conductivity [W m^−1^ K^−1^]
PUR_0	410 ± 9	38 ± 1	86.4 ± 0.6	0.0251 ± 0.0008
PUR_WS_NT	380 ± 9	42 ± 2	83.1 ± 1.1	0.0302 ± 0.0007
PUR_WS_A	360 ± 8	40 ± 3	85.9 ± 1.1	0.0293 ± 0.0009
PUR_WS_S	355 ± 8	39 ± 2	86.0 ± 0.8	0.0284 ± 0.0009

**Table 6:** Thermogravimetric analysis (TGA) results.

Sample	T_max_ [°C]			Residue at 450 °C	Residue at 600 °C	DTG [%/min]
	1st Stage	2nd Stage	3rd Stage			
PUR_0	218	315	581	48.1	21.9	0.0057
PUR_WS_NT	219	317	587	49.8	23.1	0.0055
PUR_WS_A	224	323	585	50.2	25.5	0.0056
PUR_WS_S	225	325	591	51.5	24.7	0.0056
